# Chloral-Hydrate

**Published:** 1871-01

**Authors:** William C. Bakes


### SELECTED ARTICLES.

### ARTICLE VI.

### *Chloral-Hydrate.*

By William C. Bakes.

So much has been said and written in regard to the therapeutical and chemical properties of this new remedial agent, that there may seem but little more to be said in relation to it. We shall, therefore, only mention a few of its characteristics, and suggest the most convenient modes of administering it, with a view of securing its effects as a hypnotic and anæsthetic. Mr. E. Schering, of Berlin, who has manufactured it on a large scale makes the following statement:—Chemically pure chloral-hydrate ($C_2Cl_3OH + H_2O$,) forms white acicular crystals, has a peculiar pungent odor, a somewhat bitter taste, produces, in concentrated solution, a slight irritation in the throat, fuses and sublimes readily, and keeps well in glass stoppered bottles—even in aqueous solution. In dispensing, glass, porcelain, or silver utensils are to be used.

It is readily soluble in distilled water; only after contact of the atmosphere, traces of hydrochloric acid are discernable, which must be carefully neutralized by ammonia, if the solution is to be used for sub-cutaneous injections.

In a pamphlet issued by Dr. O. Liebreich, the following formulæ are given for administering chloral-hydrate:—

℞.—Hydratis chlorali, 38 grs.;

Aq. distil,

Mucil. acaciæ, aa ℥ ss.—*M.*

*S.*—Take at one dose. (Ordinary hypnotic.)

℞.—Hydratis chlorali, 62 grs.;
Aq. distil,
Syr. aurant., aa ℥ ss.—*M.*

*S.*—A tablespoonful at night. (Ordinary hypnotic.)

℞.—Hydratis chlorali, ʒ ss;

Aq. distil, ℥ iv;

Syr. aurant,

Mucil. acaciæ, aa ℥ ss.—*M.*

*S.*—A tablespoonful every hour. (Sedative.)

Other formulæ have been used with success, and some physicians have obtained good results from the syrup of chloral.

℞.—Hydratis chlorali, 160 grs.;

Aq. distil, ʒ iv;

Syrupus, ℥ iij, ʒ ij;

Aq. flor. aurant, ʒ ij.—*M.*

*S.*—This may be given in doses of one teaspoonful or more.

A preparation less sweet than the syrup, pleasant to the taste and pleasing to the eye, is found in the elixir of chloral.

℞.—Hydratis chlorali, 160 grs.
Aq. distil, ℥ i;
Syr. aurant, rubr., ℥ iij.—*M.*

Chloral-hydrate is also used for sub-cutaneous injections, and may be dissolved in distilled water in suitatable proportions.—*Dental Times.*

